# Quantitative RT-PCR analysis of differentially expressed genes in *Quercus suber* in response to *Phytophthora cinnamomi* infection

**DOI:** 10.1186/2193-1801-3-613

**Published:** 2014-10-17

**Authors:** Ghazal Ebadzad, Alfredo Cravador

**Affiliations:** Faculdade de Ciências e Tecnologia, Universidade do Algarve, Campus de Gambelas, 8005-139 Faro, Portugal; Centre for Mediterranean Bioresourses and Food, FCT, Universidade do Algarve, Campus de Gambelas, 8005-139 Faro, Portugal

**Keywords:** Cork oak, Biotic stress, cDNA-AFLP, qRT-PCR, Defence response, Reference genes

## Abstract

cDNA-AFLP methodology was used to gain insight into gene fragments differentially present in the mRNA profiles of *Quercus suber* roots infected with zoospores of *Phytophthora cinnamomi* at different post challenge time points. Fifty-three transcript-derived fragments (TDFs) were identified and sequenced. Six candidate genes were selected based on their expression patterns and homology to genes known to play a role in defence. They encode a cinnamyl alcohol dehydrogenase2 (*QsCAD2*), a protein disulphide isomerase (*QsPDI*), a CC-NBS-LRR resistance protein (*QsRPc*), a thaumatin-like protein (*QsTLP*), a chitinase (*QsCHI*) and a 1,3-β-glucanase (*QsGlu*). Evaluation of the expression of these genes by quantitative polymerase chain reaction (qPCR) revealed that transcript levels of *QsRPc*, *QsCHI*, *QsCAD2* and *QsPDI* increased during the first 24 h post-inoculation, while those of thaumatin-like protein decreased. No differential expression was observed for 1,3-β-glucanase (*QsGlu*).

Four candidate reference genes, polymerase II (*QsRPII*), eukaryotic translation initiation factor 5A (*QsEIF-5A*), β-tubulin (*QsTUB*) and a medium subunit family protein of clathrin adaptor complexes (*QsCACs*) were assessed to determine the most stable internal references for qRT-PCR normalization in the *Phytophthora-Q. suber* pathosystem in root tissues. Those found to be more stable, *QsRPII* and *QsCACs*, were used as internal reference in the present work.

Knowledge on the *Quercus* defence mechanisms against biotic stress is scarce. This study provides an insight into the gene profiling of a few important genes of *Q. suber* in response to *P. cinnamomi* infection contributing to the knowledge of the molecular interactions involving *Quercus* and root pathogens that can be useful in the future to understand the mechanisms underlying oak resistance to soil-borne oomycetes.

**Electronic supplementary material:**

The online version of this article (doi:10.1186/2193-1801-3-613) contains supplementary material, which is available to authorized users.

## Introduction

Cork oak (*Quercus suber L.*) forests are part of Mediterranean ecosystems of Portugal, Spain, France, Italy and Morocco that, besides their high socio-economic value resulting from the exploitation of cork, play a significant ecological role. They are a permanent component of the landscape, a significant barrier against forest fires, and a source of a remarkable biodiversity due to the high habitat heterogeneity (Natividade 1950; Pausas *et al.*^2009^). Especially in Iberian Peninsula that accounts for near 90% of cork world production and where most studies have been done this ecosystem has been under the threat of a progressive decline for which the main biological contributing factor was shown to be the root parasite *Phytophthora cinnamomi,* one of the most destructive *Phytophthora* species (Brasier *et al.*^1993^; Caetano 2007; Moreira-Marcelino 2001). Despite the ecological and economic importance of cork oak, the pathosystem *Quercus-P. cinnamomi* has not received sufficient attention and molecular interaction studies are scarce. Also, rising concerns about the expected spread of *P. cinnamomi* to Northern and Eastern regions in Europe, as a consequence of climatic changes (Brasier 2003; Jung 2009) justify the investment in increasing the knowledge of this pathosystem.

Plants use various strategies to resist infection by particular pathogens. These strategies are part of the plants innate immune system. During infection plant pathogen secrete effector proteins that reprogramme host physiology and immunity for their benefit. Thus, the pathogen finds its way to counteract the basic defence responses [PAMP-triggered immunity (PTI)] (Jones and Dangl 2006). The next barrier for the pathogen to overcome is the effector-triggered immunity (ETI). This is, however, highly specific and only triggered when the plant carries the so called R genes (plant resistance genes) (Jones and Dangl 2006; Ingle *et al.*^2006^).

Little is known about the genes involved in the *Q. suber*-*P. cinnamomi* pathosystem. Current knowledge is mainly based on research of the non-host plants such as Arabidopsis. When infected with *P. cinnamomi,* this plant showed initiation of ROS generation, HR activation, lignin synthesis and callose production (Rookes *et al.*^2008^). Other changes such as callose production have also been detected in maize upon infection with *P. cinnamomi* (Hinch and Clarke 1982). However, models using genomics to study the basis of resistance to pathogens and pests are now being developed for Fagaceae species (The Fagaceae Genomic Tools Project http://www.fagaceae.org). Genomics of forest trees entered a new era boosted by the advances in increasingly fast, cost effective and reliable DNA sequencing technologies. Parallel to the development of bioinformatics tools this will allow a major breakthrough in the elucidation of the molecular mechanisms that govern biotic interactions hosted by forest trees (for comprehensive reviews see Neale and Kremer (2011) and Plomion and Fievet (2013).

Understanding the host specific interaction between *Q. suber* and the pathogen involves studying the genes expressed as a cellular response to the infection, and their role in the plant disease. To date, a number of methods have been successfully developed to identify differential gene expression in various biological systems (Frolov *et al.*^2003^; Venkatesh *et al.*^2005^). For instance, cDNA-AFLP is a reliable, rapid and genome-wide expression technique that helps gene discovery requiring no prior knowledge of gene sequences. Moreover, it is more accurate and reproducible than many other methods as it can amplify low-abundance transcripts (Breyne and Zabeau 2001), although it is necessary to confirm the obtained results by another approach such as qRT-PCR (Mills *et al.*^2001^). In a study by Coelho (2004) several *Q. suber* genes that responded to *P. cinnamomi* infection were identified and characterized. In the current work complementary study was carried out for further understanding the molecular mechanisms underlying the interaction. We report the results of cDNA-AFLP analysis to identify defence related transcripts in micropropagated clonal *Q. suber* during infection. In addition, for the first time internal reference genes were developed for qRT-PCR normalization in the *Phytophthora-Q. suber* pathosystem in root tissues and differential gene expression of selected putative defence related genes with regard to different time points of infection was performed by qPCR in *Q. suber.*

## Materials and methods

### Biological material

Micropropagated *Q. suber* plantlets (Clone SSR21) obtained from M.I. Candeias (INIAP, Instituto Nacional de Investigação Agrária e das Pescas, Lisbon) were used for transcript profiling of *Q. suber* genes.

The *P. cinnamomi* strain PA45 was isolated in the Algarve region (southern Portugal) from soil associated with declining *Q. suber* stands. Isolation and culture maintenance took place on V8 Juice agar medium as described by Horta *et al.* (2010).

To obtain zoospores, five *P. cinnamomi* isolate PA45 agar plugs were tacked from the edge of the actively growing colony and placed onto a Miracloth disc (Calbiochem), on a fresh 10% V8 Agar plate. The procedures followed to produce mycelium mats with sporangia and zoospores were described by Robinson and Cahill (2003).

Biological material used for RNA extraction was prepared by submerging micropropagated roots in a zoospore suspension (100,000 zoospores/ml) for 8, 14, 20, 26 and 32 hours placed in the dark at 25°C. Non-inoculated roots, submerged in sterile distilled water were used as control.

### Total RNA extraction

Total RNA was extracted from 50 mg of micropropagated clonal cork oak roots with the RNeasy kit from Qiagen, according to the instructions supplied by the manufacturer (Dudareva et al. 1996). Traces of DNA were removed with 2 μl DNase I (1 U/μL, Invitrogen), in the presence of 2 μl RNaseout (40 U/μL, Invitrogen) in 10 μl DNase buffer (200 mM Tris–HCl, pH 8.4, 20 mM MgCl_2_, 500 mM KCl, Invitrogen). RNA purity and integrity is essential for synthesis of full-length cDNA. Concentration of total RNA were determined by measuring the absorbance at 260 nm and the ratio of the absorbance at 260/280 nm was used to assess the RNA purity in a spectrophotometer *MBA 2000* (Perkin Elmer). RNA was considered pure when a ratio of ~2.0 was obtained. As a routine procedure the integrity of total RNA was checked by electrophoresis in a denaturing 1.2% agarose gel, stained with ethidium bromide (Sambrook and Russell 2001).

### cDNA synthesis and production of AFLP fragments

Double-stranded cDNA was synthesized from 2 μg of total RNA with the cDNA Synthesis System (Roche), with modifications to the original protocol supplied by the manufacturer. Synthesis of the first cDNA strand was initiated using the primer COE [5 'AGTGAATTCT12V (V = A, C, G)], composed of a mixture of the three oligonucleotides (Money *et al.*^1996^). Synthesis of the second cDNA strand and digestion of residual RNA were performed according to the kit protocol (Gubler 1988; Gubler and Hoffman 1983). The cDNA was purified with the Qiaquick PCR purification kit according to the protocol supplied by the manufacturer. The cDNA was removed from the column with 50 μl of Tris–HCl (10 mM, pH 8.5) and the solution was dehydrated by centrifugation under vacuum to a volume of 20 μl.

The purified cDNA was double digested with 2.5 U each of *Eco*RI and *Mse*I in the presence of 5 times reaction buffer (50 mM Tris–HCl, pH 7.5, 50 mM Mg-acetate, 250 mM K-acetate) for 2 h at 37°C. The enzymes were then inactivated for 15 min at 70°C. After inactivation of endonucleases, adapters complementary to the restriction sites *Eco*RI and *Mse*I were ligated to the digested material. Ligation was performed in the presence of 5 pmol *Eco*RI adapter (5′-AATTGGTACGCAGTCTAC-3′, 5′-CTCGTAGACTGCGTACC-3′), 50 pmol *Mse*I adapter (5′- TACTCAGGACTCAT- 3′, 5′- GACGATGAGTCCTGAG- 3′), 24 μl ligase buffer composed of ATP (0.4 mM), Tris–HCl pH 7.5, Mg-acetate (10 mM), and K-acetate (50 mM) and 1U T4 DNA ligase at 37°C for 2 h (AFLP Core Reagent Kit, Invitrogen) (Bachem *et al.*^1998^). The resulting ligated cDNA was pre-amplified with primers that hybridize to the adaptors. Pre-amplification reaction was performed in a total volume of 50 μl including 5 μl of 10 times buffer [Tris–HCl (100 mM, pH 8.3), MgCl_2_ (15 mM), KCl (500 mM)], 0.2 μl Taq DNA polymerase (5 U/μl, Boehringer Mannheim), 15 pmol of primer *Eco*RI +0 (5′-GACTGCGTACCAATTC-3′), 15 pmol of *Mse*I + 0 primer (5′-GATGAGTCCTGAGTAA-3′) and 10 μl of cDNA solution prepared above. The reaction was carried out in 28 cycles consisting of 30 s 94°C, 60 s 60°C, 60 s 72°C (Zabeau and Vos 1993).

The pre-amplified cDNA was diluted in water in a ratio of 1:10 and used as a target for the selective amplification which involved the use of combinations of *Eco*RI and *Mse*I primers containing one, two or three selective bases at the 3′ end. In total, twenty pairs of primers were used in selective amplifications. The *Eco*RI primers were radiolabelled with [γ^33^P] ATP (Redivue, Amersham Pharmacia) at the 5′ end using standard protocols (Bachem *et al.*^1998^; Zabeau and Vos 1993). Labelled selective amplification products were separated on standard 6% polyacrylamide sequencing gels. After electrophoresis, the gels were dried on filter paper (3MM paper; Whatman) and exposed to X-ray film for 30 h. The cDNA fragments were visualised by autoradiography, after positional marking the gel and the film.

### Isolation, cloning and sequencing of cDNA fragments

Those gene fragments present only in the mRNA profile of roots infected with *P. cinnamomi* and those that showed an increase or decrease in their expression were eluted from the gel. Rectangular pieces of Whatman paper were placed into tubes with the piece of excised gel in 40 μl water and kept on ice for 10 min. After hydration, the tubes were placed at 95°C for 15 min and transferred to ice. The mixture was centrifuged for a few seconds, the supernatant was collected and 5 μl of the solution were used as target DNA, in reamplification reactions (Habu and Iida 1998). These were performed with the selective primers used to generate the corresponding cDNA-AFLP profile. The re-amplified PCR products were run on a 2% agarose gel, excised and purified with the Qiaquick PCR Purification Kit (Qiagen) and cloned into the pCRII Topo vector, with the TA Cloning Kit (Invitrogen). Manufacturer’s instructions for these kits were followed throughout. The fragments were sequenced using the kit ABI PRISM BigDye Terminator Cycle Sequencing Ready Reaction (Perkin Elmer/Applied Biosystems) in an automatic sequencer ABI PRISM 377 (Perkin-Elmer) at Macrogen Company (Seoul, Korea).

### Sequence analysis

The sequences were analyzed by means of the Vector NTI 6 software™ (InforMax. Inc) and were aligned and compared through the National Centre for Biotechnology Information (NCBI) GenBank using the tblastx or blastn algorithms. Further analysis for selection of interesting cDNA-AFLP expression patterns in the time-course of the cDNA-AFLP experiment was done on the basis of significant tblastx or blastn hits sequence alignment score (e-values <0.01) (Altschul *et al.*^1997^), and homolog to genes known to play a role in disease/defence in plants. Each transcript was functionally annotated through analysis of the scientific literature. A summary of selected cDNA-AFLP fragments assigned to different functional categories is shown in Additional file 1.

### Complete sequencing of coding regions of *Quercus suber*genes

Among the candidate genes corresponding to the differentially expressed *Q. suber* fragments in response to *P. cinnamomi*, four TDFs (Clone 27–2, Clone 29–1, Clone 59–1 and Clone 60–1) which respectively have similarity with genes encoding 1,3-β-glucanase, alcohol dehydrogenase, thaumatin-like protein and chitinase were selected to identify their full-length 3′ and 5′ end.

A Full Length cDNA Library was constructed by Invitrogen using SuperscriptR Full Length cDNA Library Construction Kit. This library is guaranteed to have at least 3 × 10^6^ clones with an average insert size of 1.2 kb. Quality checks were performed and data were included in the certificate of analysis. The library screening was performed by PCR amplification.

Based on the titer of the cDNA Library on the Certificate of Analysis (1.5 × 10^6^ cfu/ml), a dilution of 1:10 was prepared and incubated at 99°C for 5 min and then placed on ice for 2 min. Five μl of dilution were used as a template in a PCR reaction which was carried out in 50 μl volumes containing 5 μl buffer 10× (200 mM Tris–HCl, pH 7.5, 1 M KCl, 15 mM MgCl_2_, DTT 10 mM, 1 mM EDTA, Tween 20, 1% v/v, Nonidet P40 1% v/v), 5 μl dNTPs (dATP, dCTP, dGTP and dTTP, 2 mM each), 0.2 μl Taq DNA polymerase (5 U/μl, Boehringer Mannheim) and with 2 μl each specific primer (10 mM) (primers specifically designed to hybridize to candidate genes for amplification of their 3′ and 5′ end) and 2 μl M13 forward and M13 reverse primers which hybridize with regions of the pDONR™ 222 vector used for cloning the double strand cDNAs in the construction of cDNA library by Invitrogen. Amplification reactions were performed using the following cycling parameters: 35 cycles of 30 s at 94°C, 30 s at 60°C and 2 min at 68°C. When no amplification was observed, reamplification was carried out by using 5 μl of the PCR product from the first reaction under the same conditions. Ten μl of each reaction was applied to a 1.5% agarose gel, an electrophoresis was run and the result analyzed. In order to amplify the coding sequences (CDS) of the above referred to selected candidate genes corresponding to the differentially expressed *Q. suber* fragments in response to *P. cinnamomi*, primers were designed based on known sequences of 3′ and 5′ ends. The fragments were cloned into vector pCRII Top Kit Top TA Cloning Invitrogen (Ausubel *et al.*^1994^; Shuman 1994). Full CDS sequences of the selected genes were analyzed in blastn available at NCBI (National Center for Biotechnology Information) (Altschul *et al.*^1997^) and multisequence alignment of nucleotide was carried out using Vector NTI7 of InforMax, Inc.

The cloned sequences were submitted to the NCBI public database (http://blast.ncbi.nlm.nih.gov/). Description of genes amplified and sequence accession numbers are shown in Table 1.

**Table 1:** **Description of target and reference genes amplified in** ***Quercus suber***

Target genes (TDF code)	Homology	Accession number	Amplicon length (bp)
1,3-beta glucanase (*QsGlu*) (clone 27–2)	*Ricinus communis* glucan endo-1,3-β-glucosidase (XM_002518884.1)	KF704744	1230
Alcohol dehydrogenase (QsADH) (clone 29–1)	*Dimocarpus longan* alcohol dehydrogenase (DQ515803.1)	KF704745	1146
Thaumatin-like protein (*QsTLP*) (clone 59–1)	*Cucumis sativus* thaumatin-like protein [(LOC101227447), (XM_004163769.1)]	KF704742	750
Chitinase (*QsCHI*) (clone 60–1)	*Fragaria vesca* subsp. *vesca* chitinase-like protein 2-like (XM_004295058.1)	KF704743	951
**Reference genes**			
RNA polymerase II (*QsRPII*)	*-*	KJ563261	954
β-tubulin (*QsTUB*)	*-*	KJ563262	1341
Eukaryotic translation initiation factor 5A (*QsEIF-5A*)	*-*	KJ563263	811
Clathrin adaptor complexes medium subunit family protein (*QsCACs*)	*-*	ID6728500	175

For the target genes, amplicon sizes were calculated based on the complete coding region sequences (CDS).

## Quantitative real-time RT-PCR (qRT-PCR)

### Biological material

In order to infect roots with mycelium of *P. cinnamomi*, germinated roots with 10 cm length and 2-week-old were infected directly by placing a 8 mm diameter V8 agar plug containing actively growing mycelium in direct contact with the root surface. Roots were collected at 6, 12, 18 and 24 hours post-inoculation (hpi). Uninfected roots at time zero were used as control for comparison of expression pattern of target genes in infected and uninfected roots. For each time point, four cork oak roots were used as biological replicates in the qPCR reaction.

### Total RNA extraction and purification

Total RNA was extracted from healthy and infected roots and trace of genomic DNA eliminated as described above. After elimination of gDNA contamination, the total RNA was purified using polyvinylpyrrolidone (PVP) which is known to remove polysaccharides, phenols and other compounds (Ainsworth 1999; Asif *et al*. 2000) and then cleaned up by phenol:chloroform extraction. This method was combined with RNeasy kit (Qiagen) for purification of RNA.

RNA concentration was determined spectrophotometrically using nanodrop ND-1000 (Thermo Scientific) and RNA quality was assessed by electrophoresis in denaturing 1.2% agarose gel.

### First strand cDNA synthesis and quality control

First strand cDNA synthesis was carried out on 500 ng total RNA from each sample, using RevertAid First Strand cDNA Synthesis Kit according to the protocol supplied by the manufacturer.

The presence of inhibitors within biological samples was assessed using as an external control, the Alien qRT-PCR Inhibitor Alert (Agilent Technologies). ALIEN RNA was added to each sample during first strand cDNA synthesis and a tube containing only the ALIEN RNA was reverse transcribed as a control following the protocol supplied by the manufacturer.

### Reference genes

Four reference genes [encoding RNA polymerase II (*QsRP II*), eukaryotic translation initiation factor 5A(*QsEIF-5A*), β-tubulin (*QsTUB*) and clathrin adaptor complexes medium subunit family protein (*QsCACs*)] were selected according to criteria described below, and their consistent PCR amplification was checked. Sequences were obtained from the Fagaceae database, Fagaceae Genome Web (The Fagaceae Genomic Tools Project http://www.fagaceae.org) and from GenBank. All the reference genes were cloned and sequenced. The sequence accession numbers are shown in Table 1.

Analysis of the stability of genes was carried out using the software GenEx (Version 5. 4. 3, MultiD Analyses, Gothenburg, Sweden), that includes both GeNorm and Normfinder programmes for selection of optimal reference genes where two parameters were defined: M (average expression stability) and stability value. Lower M or stability value indicates more stable expression, increasing the suitability of a particular gene as a control gene.

### Primer design

Gene-specific primers were designed to target sequences of *Q. suber* candidate genes and potential reference genes to amplify amplicons ranging from 86 to 229 bp (Table 2), based on conserved regions of orthologous genes from other plant families present in the GenBank database. All primers were designed using primer 3 (v. 0.4.0) software (http://frodo.wi.mit.edu/) (Rozen and Skaletsky 2000) and tested for their specificity by comparing with the genome sequences of *P. cinnamomi*, running PCR, using cDNAs of infected cork oak roots as templates and sequencing the amplicons. On the basis of these analyses the primers were judged to be specific for their target cDNA templates.

**Table 2:** **Description of primers used in RT-qPCR**

Target genes	Accession number	Primer sequence	Amplicon size (bp)	Temperature (°C)	Efficiency	r ^2^
Reverse: 5’TGAGGTTTTGAAACCCCTTG3’						
Thaumatin like protein (*QsTLP*)^a^	KF704742	*Forward*: 5’TCGGTCCCATGATAATAGAAG3’	207	55	90.4%	0.998
		Reverse: 5’TTGCCACCGGTGCAAGTAG3’				
Chitinase (*QsCHI*)^a^	KF704743	*Forward*: 5’CCGTGGTGCCTTGCCTTTG3’	177	55	96.0%	0.994
		Reverse: 5’GCATCATGTGCTGAAGGCTG3’				
1,3-β-glucanase (*QsGlu*)^a^	KF704744	*Forward*: 5’CACTCTCACTGGTTGTCTTG3’	175	55	93.6%	0.996
		Reverse: 5’CGGGGCTATGCAGCTGGTC3’				
Cinnamyl alcohol dehdrogenase 2 (*QsCAD2*)^b^	AY362455	*Forward*: 5’AGCGGGTAGTTGTGACATC*C*3*’*	229	55	93.8%	0.998
		Reverse: 5’AGAGGACCAATCACCATTGC3’				
Protein disulphide isomerase (*QsPDI*)^b^	AY525090; AJ316614	*Forward*: 5’GCCACTGGGAAGGTTGTGAC3’	221	55	94.9%	0.998
		Reverse: 5’GTTTTGCCTTCAAGCTCAGG3’				
CC-NBS-LRR resistance protein (*QsRPc*)^b^	GU289638	*Forward*: 5’GTTGCAACAACCCACTTCTC3’	182	55	90.0%	0.994
		Reverse: 5’TGAGGTTTTGAAACCCCTTG3’				
**Reference genes**		**Primer sequence**	**Amplicon size (bp)**	**Temperature (°C)**	**Efficiency**	**r** ^**2**^
RNA polymerase II (*QsRPII*)	KJ563261	*Forward* : 5’GACATAGATCCCGTTACCCA3’	168	55	92.3%	0.999
		Reverse: 5’TTTGATTGCACCAGTAGATTC3’				
β-tubulin (*QsTUB*)	KJ563262	*Forward*: 5’GCTCACTACCCCAAGCTTT3’	187	55	90.5%	0.999
		Reverse: 5’GGAACCTCTGGAGGTTAAA3’				
Eukaryotic translation initiation factor 5A (*QsEIF-5A*)	KJ563263	*Forward*: 5’GCCATGTCCGACGAGGAG3’	86	57	90.2%	1
		Reverse: 5’CGGATGGTTCCGGCTTGC3’				
Clathrin adaptor complexes medium subunit family protein (*QsCACs*)^c^	ID6728500	*Forward*: 5’TCTGGGAGAAGAGTGGCTACA3’	175	55	96.3%	0.993
		Reverse: 5’GAGCCACCATTCAAATCCT3’				

^a^From cDNA-AFLP candidate genes; ^b^from Coelho(2004); ^c^from Marum *et al*. (2012).

PCR efficiencies and coefficients of primers were determined with duplicated standard curves generated from 10-fold dilution series (from 1 ng to 1 fg) of purified PCR fragments as templates. Amplification efficiencies for each gene and coefficients of determination (r^2^) between concentration and signal intensity were calculated from the slopes of the standard curves for all genes.

### QPCR amplification

Quantitative RT-PCR reactions were performed in an iQ5 multicolour Real-Time PCR Detection System (Bio-Rad) using SsoFast™ Evagreen® supermix kit (Biorad). For each experimental sample 2 μl of diluted cDNA (2.5 ng), 7.5 μl of SsoFast™ Evagreen® supermix, 1 μl of forward primer and 1 μl of reverse primer (final concentration 50 nM each primer) in a final volume of 15 μl were used. The following standard thermal cycling profile was used: 95°C for 10 min; 30 s at 95°C, 40 cycles of 1 min at optimal annealing temperature for each pair of primer and 30 s at 72°C. To confirm that the signals were the result of a single amplified product, melting curve analysis was performed after the run by increasing the temperature of 0.5°C by cycle from 65°C to 95°C. To estimate variation in the technique, two technical replicates were carried out for each biological replicate and four biological replicates were used for each time point of infection (6, 12, 18 and 24 hpi) and non-infected roots (time 0).

Expression levels were determined as the number of cycles (Ct) needed for the amplification to reach a threshold fixed in the exponential phase of PCR reaction (Walker 2002). For each repetition of samples in every run, the level of gene expression was normalized to that of *QsRP II* and *QsCACs* by means of “Ct of target—Ct of reference gene” formula namely ΔCt. Furthermore, the mean of ΔCt values for each target gene was then normalized to the expression of infected samples with non-infected samples to find ∆∆Cq. This accounted for any effects associated with the experimental procedure and was expressed as the ratio of the targeted ∆Cq expression to the non-targeted ∆Cq expression method described by Applied Biosystems.

*QsRPII* and *QsCACs* housekeeping genes were chosen based on geNorm and NormFinder algorithm available in GenEx (Version 5.4.3, MultiD Analyses, Gothenburg, Sweden) for normalization of real-time quantitative RT-PCR data.

### Statistical analysis

Temporal expression data was analyzed using SPSS (SPSS ver. 20). One-way analysis of variance (ANOVA) with Tukey's post hoc tests was used to reveal significant differences.

## Results and discussion

### Transcript profiling of *Q. suber*genes in response to *Phytophthora cinnamomi*infection

**Figure 1:**
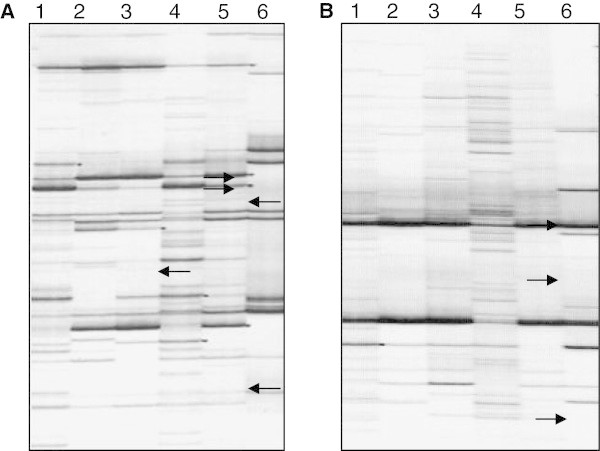
**Comparing cDNA-AFLP fragments derived from micropropagated** ***Quercus suber*** **roots, before and after inoculation with zoospores from** ***Phytophthora cinnamomi*** **.** The examples show selective amplification with primer combinations *Eco*RI*-*ACC*/Mse*I-CTA **(A)** and *Eco*RI*-*ATG*/Mse*I-CAA **(B)** in host roots cells at 0, 8, 14, 20, 26 and 32 h post challenge (from 1 to 6, respectively). Examples of differentially expressed fragments are indicated by arrows in black.

The molecular mechanisms underlying *Quercus-*soil-borne pathogens interaction is largely unknown. The cDNA-AFLP methodology is a simple, rapid and very powerful tool for discovery of novel genes in organisms especially when little is known about the genome sequences (Breyne and Zabeau 2001). In this study, a cDNA-AFLP analysis of differential gene expression in clonal cork oak roots response to *P. cinnamomi* was conducted. Selective amplification with 20 primer combinations allowed the visualization of 53 transcript-derived fragments (TDFs) with 47–485 bp size (Figure 1). These fragments were sequenced and the sequences further analyzed by blastn and blastx. A homology search showed that among the determined sequences, 66% were homologous to known sequences while 26.5% were homologous to genes with unknown function and 7.5% had no matches with databases. Among these TDFs, 92.5% were related to *Q. suber* and 7.5% were attributed to *P. cinnamomi.*

A summary of most interesting selected cDNA-AFLP fragments and their annotation is shown in Additional file 1.

TDFs (Clone 27–2, Clone 59–1 and Clone 60–1) which have direct blast hits to known annotated genes that play a role in disease/defence in plants were selected for the following analysis. These derived from genes that encode 1,3-beta glucanase, thaumatin-like protein and chitinase, respectively. They represent various functions assigned to proteins deduced from these genes such as activation of defence signalling pathways, anti-fungal activity [ex. 1,3-β-glucanase, chitinase (Mauch and Staehelin 1989) and thaumatin like protein (Kim *et al.*^2009^)].

In order to accurately assess differential expression patterns of genes revealed by cDNA-AFLP, qRT-PCR evaluation of these and of additional three genes was further conducted. As a first step, to avoid bias, reliable reference genes to be used as internal controls were judiciously selected and validated.

### Gene expression analysis by qRT-PCR and selection and validation of reference genes

In a previous study*, QsCACs* and *actin* were described as good reference genes in *Q. suber* (Marum *et al.*^2012^). They were evaluated among several others, in several tissues that did not include roots. Whenever analysing transcript expression levels by RT-qPCR in different tissues and under different conditions a new evaluation of reference genes is required. This is the case of *Q. suber* roots challenged with *P. cinnamomi* infection that involves substantial changes in the process under study compared with the above study.

*QsRPII, QsCACs, QsTUB* and *QsEIF-5A* were selected based on their previous use as internal controls in gene expression studies of species such as *Q. suber*, *Prunus persica* and *Musa acuminata* (Marum *et al.*^2012^; Tong *et al.*^2009^; Chen *et al.*^2011^) and on their consistent PCR amplification. The stability of these potential reference genes was analyzed using GenEx (Version 5.4.3, MultiD Analyses, Gothenburg, Sweden). M values of all selected reference genes were lower than 1, attesting they are stably expressed housekeeping genes in our samples. *QsRPII* and *QsCACs* were the two most stable genes with an equal M value of 0.247. *QsTUB* and *QsEIF-5A* were the third and the fourth most stable genes, with M values of 0.483 and 0.744, respectively.

In the relative quantification of gene expression, the use of multiple reference genes that are stable under a given experimental condition was proven to be an efficient method for the normalization of RT-qPCR (Migocka and Papierniak 2011; Demidenko *et al.*^2011^; Die *et al.*^2010^; Xu et al. 2011; Expósito-Rodríguez *et al.*^2008^; Schmidt and Delaney 2010). In order to determine how many reference genes should be included in the normalization process, Acc.SD of the four genes were calculated by NormFinder algorithm available in GenEx (Version 5.4.3, MultiD Analyses, Gothenburg, Sweden). The optimal number of reference genes is indicated by the lowest value for the Acc.SD. This was found when using two reference genes among the four reference genes. The improvement of using two reference genes (Acc.SD = 0.1087) instead of only one (Acc.SD = 0.1234) is very small. Nevertheless, in order to avoid the weakness of using one single reference gene and for more accurate normalization of the qRT-PCR data (Die and Rowland 2013), the combination of two control genes *QsRPII* and *QsCACs* according to M-value and Acc.SD values was used for normalization of data. These results are valuable data for future studies on gene expression in this or eventually, other pathosystems involving oaks.

The specificity of PCR amplification of each primer pair was confirmed by sequencing of PCR products and by melting curve analysis during real-time PCR. Amplification efficiencies during different PCR runs varied between 90% and 96.3%, and the r^2^ values varied from 0.993 to 1 as described in Table 2. These make the normalized expression ratios reliable and accurate.

The relative expression analyses of six genes of *Q. suber* by qRT-PCR were conducted at 0, 6, 12, 18 and 24 hpi in order to validate the cDNA-AFLP results. Gene expression was calculated as the ratio between target gene transcript number and RNA polymerase II (*QsRPII*) and clathrin adaptor complexes medium subunit family protein (*QsCACs*) transcripts as reference genes. Defence-related genes coding for thaumatin-like protein (*QsTLP*), chitinase (*QsCHI*), 1,3-β-glucanase (*QsGlu*), a cinnamyl alcohol dehydrogenase2 (*QsCAD2*), a protein disulphide isomerase (*QsPDI*) and a NBS-LRR resistance protein (*QsRPc*) were chosen for evaluation of their expression by qRT-PCR. The latter three genes have been previously identified by cDNA-AFLP and characterized (Coelho 2004). We decided to select them due to their potential important role in the defence response of cells against plant pathogens as shown in previous studies (Coll *et al.*^2011^; Meyers *et al.*^1999^, ^2003^; Kohler *et al.*^2008^; Soriano *et al.*^2005^; Radwan *et al.*^2008^; Chandra *et al.*^2007^; Hammond-Kosack and Jones 1997; Milcevicova *et al.*^2010^; Vieira Dos Santos and Rey 2006; Lamb and Dixon 1997; Garin *et al.*^2001^; Muller-Taubenberger *et al.*^2001^; Augusto *et al.*^2002^; Nathan and Hibbs 1991; Ray *et al.*^2003^; Nwugo *et al.*^2013^).

**Figure 2:**
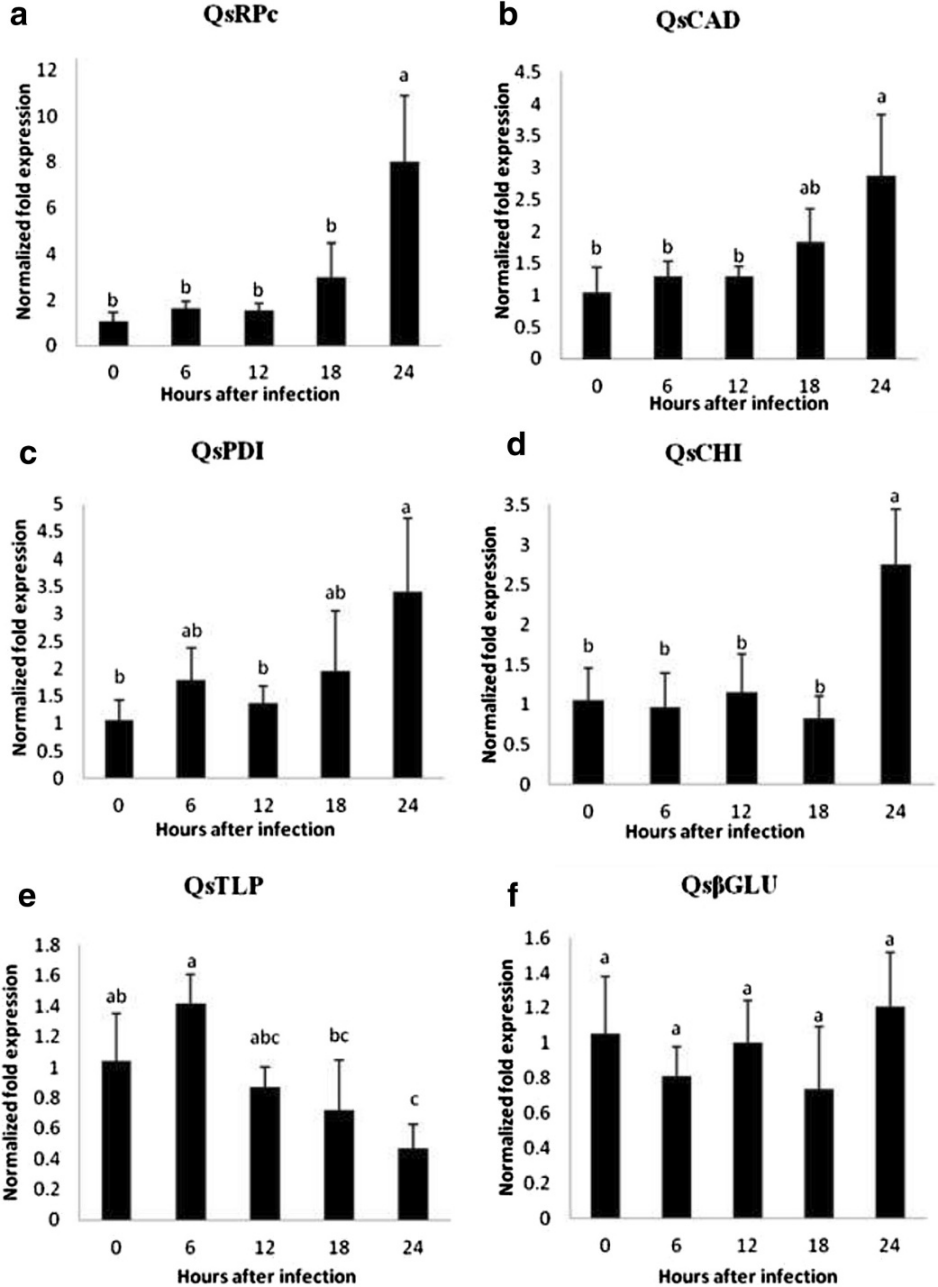
**qRT-PCR expression profiles of six** ***Quercus suber*** **candidate genes, CC-NBS-LRR resistance protein (** ***QsRPc: a*** **), cinnamyl alcohol dehydrogenase2 (** ***QsCAD2: b*** **), protein disulphide isomerase (** ***QsPDI*** **: c), chitinase (** ***QsCHI: d*** **), thaumatin-like protein (** ***QsTLP: e*** **), and 1,3-β-glucanase (** ***QsGlu: f*** **).** *Q. suber* roots were infected with *P. cinnamomi* in a 24 h time course of gene expression. Gene expression (y-axis) was calculated as the ratio between the transcript number of the *Q. suber* target genes and the geometric mean of transcript copies of selected housekeeping genes [RNA polymerase II (*QsRP II*) and clathrin adaptor complexes medium subunit family protein (*QsCACs*)]. Statistically significant differences (P < 0.05) between infected and non-infected (control) plants are indicated with different letters. Error bars represent the standard deviation of four biological replicates.

The transcript profiles of *QsRPc*, *QsCAD2*, *QsPDI* and *QsCHI* showed a significant up-regulation following inoculation (*P* < 0.05) (Figure 2a, b, c and d). The most significant up-regulation was observed in the transcript profile of *QsRPc* gene which showed a 7.54-fold higher expression level at 24 hpi as compared to its expression in healthy roots at time 0. *QsCAD2* and *QsPDI* exhibited significant increases by 2.74-fold and 3.22-fold, at 24 hpi, respectively, as opposed to the uninfected sample (Figure 2b and c). *QsCHI* expression showed a constant expression pattern up to 18 hpi, followed by a significant up-regulation of 2.6-fold at 24 hpi, compared to time 0 (Figure 2d). In contrast the expression of *QsTLP* was down regulated, exhibiting a significant decrease at 24 hpi (Figure 2e). No significant difference in expression of *QsGlu*, between infected and healthy roots was observed (Figure 2f). QRT-PCR confirmed the expected up-regulation for *QsRPc*, *QsCAD2*, *QsPDI* and *QsCHI* and the down-regulation for *QsTLP* showed by cDNA-AFLP. On the contrary, unexpectedly, *QsGlu* showed no differential expression by qRT-PCR being probably a false positive in cDNA-AFLP analysis.

R proteins have been attributed the function of recognizing specific pathogen effectors, assuming the role of the so called effector-triggered immunity (ETI) resulting often in the induction of hypersensitive response (HR) and programmed cell death (PCD) (Coll *et al.*^2011^).

Resistance gene families have been evaluated in numerous plants including *A. thaliana* (Meyers *et al.*^1999^, ^2003^), *Populus trichocarpa* (Kohler *et al.*^2008^) and *Prunus armeniaca L.* (Soriano *et al.*^2005^). The majority of plant disease resistance genes identified to date code to proteins belonging to a limited number of structural classes, of which those containing nucleotide binding site (NBS) motifs are the most common. Classification of these proteins is based primarily on predicted domains and motifs. (Hulbert *et al.*^2001^).

In the present work one of the most important identified gene is the one encoding a resistance protein (*QsRPc*) up-regulated upon infection by *P. cinnamomi*. This gene was shown to be 4275 bp long, to comprise an ORF encoding a polypeptide of 1425 amino acid (GenBank accession number-GU289638) and shows all the motifs characteristic of a CC-NBS-LRR resistance gene subclasse (Coelho 2004). The notable 7.5-fold expression increase of this gene at 24 hpi when compared with the control, suggests the expressed protein acts as a putative host target to effectors produced by the pathogen *P. cinnamomi* during infection and results in defence response in *Q. suber* against the pathogen attack. CC-NBS-LRR genes are part of a large family in which some members can be down-regulated as illustrated in the interaction *Q. suber*-*Pisolithus tinctorius* and *Q. robur- Microsphaera alphitoides* reported by Sebastiana *et al.*(2014) and Kurth *et al.* (2014), respectively.

It is known that the defence system in plants has multiple forms; one of these forms is the activation, during infection, of the transcription of genes involved in the synthesis of phenolic compounds as precursors for suberin and lignin production (Chandra *et al.*^2007^; Hammond-Kosack and Jones 1997; Milcevicova *et al.*^2010^). Lignin and suberin which are used as structural components of plant cell walls act as a physical barrier blocking the entry of the pathogen. Enzymes, like peroxidases, cinnamyl alcohol dehydrogenases and phenylalanine ammonia-lyases, participate in the phenylpropanoid metabolism. Therefore, the up-regulation of the expression of cinnamyl alcohol dehydrogenase2 (*QsCAD2*) upon infection by *P. cinnamomi* could be the manifestation of its participation in this metabolism as a response to the attack by the oomycete. The gene encoding *QsCAD2* was previously identified in cork oak (GenBank accession no: AY362455). This gene contains the catalytic domain for binding of zinc ion and the binding domain for NADP cofactor (Coelho 2004). We report now accurately its up-regulation by qRT-PCR, showing its role in the defence response of cork oak.

The protein disulphide isomerase gene (*QsPDI*) was shown to be 3098 bp long and to comprise an ORF encoding a polypeptide of 507 amino acid (AY525090; AJ316614) containing the WC(G/P)PC motif and three repeated thiorredoxin domains (Coelho 2004). *QsPDI* expression increased 24 h after infection, suggesting this gene can be activated by oxidation or reduction and/or isomerisation of cysteine residues, transcription factors and other proteins involved in the cellular response to the oxidative burst that occurs after pathogen invasion (Vieira Dos Santos and Rey 2006). Reactive oxygen species (ROS) induced in plants upon infection usually cause damage to pathogens. In addition, ROS (and redox signals) behave as a second messenger participating in different cellular pathways in the host-pathogen interaction (Lamb and Dixon 1997). The regulation of its production by Nox family NADPH oxidase enzyme (Augusto *et al.*^2002^; Nathan and Hibbs 1991) is mediated by PDI, a redox sensitive protein that is induced during pathogen infection. The importance of the role of PDI in the control of different steps of infection processes is supported by its well described participation in the pathogen attachment-internalization and antigen processing in the endoplasmic reticulum (ER)/phagosome (Garin *et al.*^2001^; Muller-Taubenberger *et al.*^2001^). Rapid induction of PDI in wheat after fungal inoculation during early response has been previously reported (Ray *et al.*^2003^). Also, in another study, PDI were up-regulated in lemon plants in response to Citrus huanglongbing (HLB) disease (Nwugo *et al.*^2013^).

Chitinases are hydrolytic enzymes which can play a role in weakening and decomposing of fungal cell walls (Selitrennikoff 2001; Van Loon and Van Strien 1999). Chitinase accumulates around fungal hyphal material *in planta* (Benhamou *et al.*^1990^; Wubben *et al.*^1992^). Alone or in a combination with 1,3-β-glucanase the enzyme was shown to cause the lysis of hyphal tips resulting in *in-vitro* inhibition of various fungi growth (Broglie *et al.*^1991^; Mauch and Staehelin 1989; Schlumbaum *et al.*^1986^). Furthermore, transgenic plants with induced chitinase levels are able to reduce the damage caused by the pathogens (Broglie *et al.*^1991^). Moreover, it was reported that chitinases inhibit zoospore germination in *Phytophthora* (Young and Hwang 1996).

Yan *et al.* (2008) reported the existence of chitin in cell walls of *Pythium aphanidermatum* although at half the extent of fungal cell walls. Furthermore, Sharma *et al.* (2009) reported that in taro plant inoculated with *P. colocasiae* there was induction of a putative chitinase gene. This suggests the importance of chitinase in the resistance against this oomycete. It is also well documented that plant chitinases are both directly or indirectly involved in plant resistance towards many fungal pathogens (Schlumbaum *et al.*^1986^). Sequence analysis revealed a coding region with a size of 951 bp encoding 316 amino acid (GenBank accession no: KF704743) with 82% homology to *Fragaria vesca subsp. vesca* chitinase-like protein 2-like. Within a family of 19 chitinases most are found mainly in plants but some are found in bacteria as well. Structural analysis reveals that this family together with a family comprising 46 chitosanases is similar to several lysozymes (Robertus and Monzingo 1999). The significant up-regulation of *Q. suber* chitinase during *P. cinnamomi* root infection suggests the importance of this enzyme in plant defence against the pathogen attack and constitutes probably, an effective response to lessen *P. cinnamomi* damage.

Systematic Acquired Resistance (SAR) in plants is found to correlate with induction of a set of putative defence genes including PR proteins (Linthorst 1991). One group of PR proteins (PR-5) is designated as thaumatin-like proteins based on the proteins extensive homology with thaumatin, a sweet-tasting protein from *Thaumatococcus daniellii* (Cornelissen *et al.*^1986^). In *Arabidopsis,* SAR has been demonstrated to occur against pathogens and this resistance was correlated with induction of PR proteins including a thaumatin-like protein (Uknes *et al.*^1992^). The thaumatin-like proteins purified from different plant systems have been shown to inhibit the growth of fungal pathogens *in vitro* (Vigers *et al.*^1991^; Woloshuk *et al.*^1991^) and among the defence-related proteins, thaumatin-like proteins (TLPs) are well known to function as anti-fungal proteins (Kim *et al.*^2009^). The cDNA-AFLP analysis revealed a gene coding for thaumatin-like protein (GenBank accession no: KF704742) from *Q. suber*. A 750 bp coding sequence region that showed 80% sequence similarity with *Cucumis sativus* thaumatin-like protein [(LOC101227447), mRNA (XM_004163769.1)] was resolved. TLPs are three-domain, crescent-fold structures with either an electronegative, electropositive, or neutral cleft occurring between domains I and II. The strong electronegative character of the cleft is presumably responsible for the antifungal activity of this protein (Leone *et al.*^2006^). Some TLPs have the capacity to hydrolyze the 1,3-beta glucans commonly found in fungal walls (Grenier *et al.*^1999^). Most typical TLPs described to date generally possess 16 conserved cysteine residues that form eight disulfide bonds (Ghosh and Chakrabarti 2008). Interestingly, contrary to reports from prior studies, the present study showed a general reduction in the production of the *Q. suber* thaumatin-like protein within 24 hours post-infection. This suggests that *P. cinnamomi* might suppress this host defence response processes to facilitate invasion, or alternatively that the induction of the production of this protein does not take place within 24 hpi.

Plants produce a variety of hydrolytic enzymes including chitinases, proteases and also glucanases, in defence against pathogens (Stintzi *et al.*^1993^). 1,3-β-glucanases (glucan endo-1,3-glucosidases, β-1,3-Gs) are hydrolytic enzymes that catalyse the hydrolysis of 1,3-ß-D-glucosidic linkages in β-1,3-glucans in the pathogen cell wall; they are found in bacteria, fungi, metazoan (Bachman and McClay 1996) and viruses (Sun *et al.*^2000^). In addition they were shown to be involved in cell division, pollen development and tube growth, regulation of plasmodesmata signalling, cold response, seed germination, and maturation (Hoj and Fincher 1995; Leubner-Metzger 2003, Leubner-Metzger and Meins 1999). Thus, β-1,3-Gs play major roles in developmental plant stages. The sequence of the gene coding for a 1,3-β-glucanase protein (GenBank accession no: KF704744) revealed a region with 1230 bp encoding 409 amino acid and showed 74% homologous to *Ricinus communis* glucan endo-1,3-β-glucosidase. In this study, 1,3-β-glucosidase gene expression, contrary to expectations, showed not to be significant in response to the *P. cinnamomi* infection. Therefore we can conclude that this β-1,3-Gs is related with developmental stages of the plant and not with the response to the pathogen.

We could not exclude, however, that given their membership in large gene families, the down-regulation of TLP and the lack of differential expression of β-1,3-Gs observed in the present study are due to the fact that we targeted paralogous sequences not involved in a defence response within the 24 hpi.

It is interesting to read our findings in light of the most recent transcriptome studies on *Q. suber.* Sebastiana *et al*. (2014) reported recently the differential expression of *Q. suber* genes comparing transcriptome of ectomycorrhizal (EM) colonized roots with nonsymbiotic roots. Proteins of the NBS-LRR family (namely, CC-NBS-LRR resistance gene subclasse) were found to be the most abundant products of differentially expressed genes (namely, up-regulated). Their involvement in a response to EM colonization, suggests that not only they detect pathogens like *P. cinnamomi* as observed in our work but also fungi engaged in a mutualistic association.

However, *QsCAD2, QsPDI, QsTLP, QsCHI* and *QsGlu* were not found to be differentially expressed as a consequence of the EM colonization.

As a matter of fact it was not expected to observe up-regulation of chitinase and 1,3-β-glucanase as these enzymes are involved in the lysis of hyphal tips and would inhibit mycorrhization. The same applies to TLPs whose activity against fungi proteins has been described (Kim *et al.*^2009^). Also, cinnamyl alcohol dehydrogenases appears not to participate in the phenylpropanoid metabolism suggesting mycorrhization does not trigger cell wall reinforcement that would oppose hyphae penetration and the success of colonization. Nevertheless, it was expectable to observe *QsPDI* differential expression as the enzyme is involved in the response to the oxidative burst that was shown to occur in EM establishment, at least in *Castanea sativa* (Baptista *et al.*, 2007). Also, Tarkka *et al.* (2013) found chitinase to be down-regulated in *Q. robur* mycorrhizal roots and genes related to phenylpropanoid metabolism depleted. Overall, these studies show that plant defences are attenuated in the mature oak EMs.

Interestingly, beneficial rhizobacteria appear to stimulate plant defences as it was recently shown in *Q. robur* challenged with *M. alphitoides* (Kurth *et al.*, 2014). In this study, a large number of defence-related genes were differentially expressed in plants co-inoculated with a nonpathogenic *Streptomyces sp*. strain AcH 505 and oak powdery mildew. An identified CC-NBS-LRR gene was down-regulated compared to plants non-inoculated or inoculated either with the bacterium or with the fungus; no other genes from this family were reported. This observation and the above results show the complexity of the mechanisms in which this family of resistance genes appear to be involved. Cinnamyl alcohol dehydrogenase and one chitinase were up-regulated at the same level in plants inoculated either with AcH 505 or oak powdery mildew or co-inoculated as compared to non-inoculated, in agreement with our results. No mention is made to the other genes studied in the present work.

In this study, we provide a brief insight into the expression pattern of some putative cork oak defence related genes during root infection by *P. cinnamomi*. The identification of these genes is a useful contribution to design and implement screening strategies aiming at the future development of breeding lines. Furthermore, in this study and for the first time a set of potential reference genes were analyzed and validated in root tissues for qRT-PCR normalization in the *Phytophthora-Q. suber* pathosystem. This is an important issue as the choice of suitable reference genes in any new experimental system is absolutely crucial in qRT-PCR.

## Electronic supplementary material

Additional file 1: **Similarity of selected differentially expressed TDFs in the** ***Quercus suber***–***Phytophthora cinnamomi*** **interaction with sequences detected using blastn/tblastx (Altschul** ***et al*** **.** 1997 **) and their annotation.** (DOCX 20 KB)
