# Supplementary material for: Quantitative RT-PCR analysis of differentially expressed genes in Quercus suber in response to Phytophthora cinnamomi infection
Source: Springerplus. 2014 Oct 17;3:613. doi: 10.1186/2193-1801-3-613 (PMC4221558; doi:10.1186/2193-1801-3-613)
Supplement: Supplementary file 1 — Additional file 1: Similarity of selected differentially expressed TDFs in the Quercus suber–Phytophthora cinnamomi interaction with sequences detected using blastn/tblastx (Altschul et al . 1997 ) and their annotation. (DOCX 20 KB) [file 40064_2014_1333_MOESM1_ESM.docx]

**Additional file 1.** Similarity of selected differentially expressed TDFs in the *Quercus suber*–*Phytophthora cinnamomi* interaction with sequences detected using blastn/tblastx (Altschul *et al*. 1997) and their annotation.

| Code of the cDNA-AFLP fragment | Sample^2^ | Size (bp) | Accession number of blast hits | Homology | Annotation | %Homology  (tblastx/blastn) | E-value |
| --- | --- | --- | --- | --- | --- | --- | --- |
| C3 (Clone 3-1) | HR-32 | 199 | DQ465765.1 | *Sesbania drummondii* clone SSH-12_01_B03_T3 mRNA | Response to stimulus | 95% in 41aa out of 43aa | 6e^-34^ |
| C14 (Clone 14-1) | HR-32 | 383 | NM_120041.3 | *Arabidopsis thaliana* calcium-binding EF hand family protein (AT4G38810) mRNA, complete cds | Signal transduction | 79% 58aa out of 73aa | 4e^-30^ |
| C15 (Clone 15-1) | HR-32 | 188 | FJ410447.1 | *Betula luminifera* cellulose synthase (CesA5) mRNA, complete cds | Metabolism | 97% 48aa out of 49aa | 2e^-28^ |
| C19 (Clone 19-2) | IR-8 | 381 | DQ677588.1 | *Prunus spinosa* S-RNase (S-RNase) gene, S-RNase-9 allele and S-locus F-box protein (SFB) gene, SFB-9 allele, partial cds | Metabolism | 82% in 37nt | 0.10 |
| C25 (Clone 25-1) | HR-32 | 352 | AY274259.1 | *Cucumis sativus* tyrosine kinase mRNA, partial cds | Signal transduction | 54% in 23aa out of 42aa | 1e^-13^ |
| C27 (Clone 27-2)^1^ | HR-32 | 202 | BT000048 | *Arabidopsis thaliana* putative β-1,3-glucanase precursor, putative  (At1g32860) mRNA, complete cds | Defence response/growth and development | 60% in 39aa out of 65aa | 2e^-18^ |
| C29 (Clone 29-1)^1^ | IR-32 | 380 | NM_128167.3 | *Arabidopsis thaliana* glycinedehydrogenase (decarboxylating)  (ATGLDP2) mRNA, complete cds | Photosynthesis, energy  and survival | 88% in 111aa out of 126aa | 5e^-61^ |
| C30 (Clone 30-2) | IR-32 | 190 | EEF43218.1 | Anthranilate N-benzoyltransferase protein, putative (*Ricinus Communis*) | Defence response | 65% in 38aa out of 58aa | 7e^-12^ |
| C35 (Clone 35-2) | IR-20 | 157 | NP_180174.1 | Bet v I allergen family protein [*Arabidopsis thaliana*] | Defence response | 75% in 34aa out of 45aa | 4e^-11^ |
| C43 (Clone 43-1) | IR-26 | 75 | FJ656201.1 | *Quercus ilex* subsp. *rotundifolia* AFLP fragment Rot10 sequence | Similar to retrotransposon | 69% in 16aa out of 23aa | 0.093 |
| C44 (Clone 44-2) | IR-26 | 64 | NM_125596.3 | *Arabidopsis thaliana* diphthamide synthesis DPH2 family protein  (AT5G62030) mRNA, complete cds | Elongation | 80% in 50aa out of 62aa | 0.46 |
| C47 (Clone 47-1) | IR-20 | 89 | NM_125596.3 | *Glycine max* mRNA for PDR-like ABC-transporter (pdr12 gene) | Transport | 89% in 25aa out of 28aa | 3e^-08^ |
| C51 (Clone 51-1) | IR-20 | 116 | XM_002330233.1 | *Populus trichocarpa* 2-oxoglutarate-dependent dioxygenase (2OGox2), mRNA | Oxygenases and metabolism | 81% in 18aa out of 22aa | 3e^-04^ |
| C56 (Clone 56-1) | IR-26 | 76 | NM_117063.1 | *Arabidopsis thaliana* avirulence-responsive family protein–  avirulence-induced gene (AIG1) family protein (AT4G09950) mRNA, complete cds | Defence response | 90% in 28nt | 1.9 |
| C59 (Clone 59-1)^1^ | HR-32 | 59 | NM_113307.2 | *Arabidopsis thaliana* disease resistance-responsive family protein (AT3G24020) mRNA, complete cds | Defence response | 87% in 28nt | 4.2 |
| C60 (Clone 60-1)^1^ | IR-26 | 308 | XM_002276527.2 | *Vitis vinifera* chitinase-like protein 2-like (LOC100261982) | Defence response | 86% in 84aa out of 98aa | 2e^-57^ |

^1^ TDFs were selected to complete the coding region sequences (CDS) and gene expression analysis (with the exception that C29 was not used for qRT-PCR analysis)

^2^ IR: infected roots; HR: healthy roots (control). Figures refer to the time in hours at which relevant differences were observed between IR and HR. For example, HR-32 means that C59 was not observed within 32 h (or faintly observed) in IR and that the corresponding fragment was excised from the HR sample at that time.
